# Localized poïkiloderma

**DOI:** 10.11604/pamj.2017.27.94.12959

**Published:** 2017-06-07

**Authors:** Amina Kissou, Badr Eddine Hassam

**Affiliations:** 1 Service de Dermatologie, Centre Hospitalier Universitaire Ibn Sina, Rabat, Maroc

**Keywords:** Mycosis, poikyloderma, fongoide

## Image in medicine

A patient aged 58 years, diabetic, consulted for the appearance of a lesion on the left thigh that evolved for 2 years. The clinical examination found a plaque, with digital edges, hyper-pigmented center dotted with a few areas of skin atrophy and a hypochromic periphery. The lesion was not infiltrated, mildly pruriginous and measured 10 cm of major axis (Panel A). There were no adenopathies. Histology demonstrated an orthokeratosic hyperkeratosis. The dermis was the site of an inflammatory infiltrate of increased lymphocytes of incised size. This infiltrate was arranged in a linear manner, producing the appearance in “Indian file” (Panel B). The cells were CD3+, CD4+ and CD8- (Panel C and D). The diagnosis of mycosis fungoides in its poïkilodermic form was retained. An extension report was without anomaly. The lymphoma was classified T1N0M0. Treatment with dermocorticoid associated with phototherapy “UVB-type” allowed complete remission with a follow-up of 2 years.

**Figure 1:**
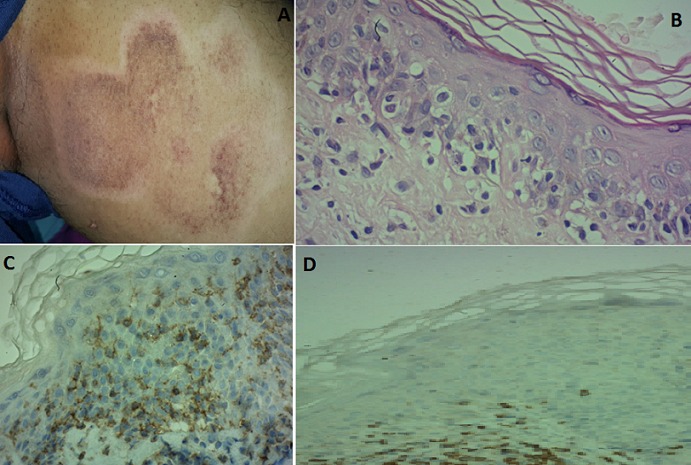
(A): plaque on the left tight: digitiform edges with dyschromatosis; (B) epidermotropism HEx40; (C) CD4+; (D) CD8–

